# The aftereffect of the ensemble average of facial expressions on subsequent facial expression recognition

**DOI:** 10.3758/s13414-021-02407-w

**Published:** 2022-02-15

**Authors:** Kazusa Minemoto, Yoshiyuki Ueda, Sakiko Yoshikawa

**Affiliations:** 1 Kokoro Research Center, Kyoto University, 46 Shimoadachi, Yoshida, Sakyo 606-8501 Kyoto, Japan; 2 Faculty of Art and Design, Kyoto University of the Arts, 2-116 Kitashirakawa Uryuyama, Sakyo, Kyoto, 606-8271 Japan

**Keywords:** Facial expression, Ensemble perception, Priming, Adaptation, Aftereffect

## Abstract

An ensemble or statistical summary can be extracted from facial expressions presented in different spatial locations simultaneously. However, how such complicated objects are represented in the mind is not clear. It is known that the aftereffect of facial expressions, in which prolonged viewing of facial expressions biases the perception of subsequent facial expressions of the same category, occurs only when a visual representation is formed. Using this methodology, we examined whether an ensemble can be represented with visualized information. Experiment 1 revealed that the presentation of multiple facial expressions biased the perception of subsequent facial expressions to less happy as much as the presentation of a single face did. Experiment 2 compared the presentation of faces comprising strong and weak intensities of emotional expressions with an individual face as the adaptation stimulus. The results indicated that the perceptual biases were found after the presentation of four faces and a strong single face, but not after the weak single face presentation. Experiment 3 employed angry expressions, a distinct category from the test expression used as an adaptation stimulus; no aftereffect was observed. Finally, Experiment 4 clearly demonstrated the perceptual bias with a higher number of faces. Altogether, these results indicate that an ensemble average extracted from multiple faces leads to the perceptual bias, and this effect is similar in terms of its properties to that of a single face. This supports the idea that an ensemble of faces is represented with visualized information as a single face.

**Supplementary Information:**

The online version contains supplementary material available at 10.3758/s13414-021-02407-w.

Facial expression is one of the most important social cues for communicating with others, and many studies have explored its recognition system. Recently, studies have suggested that we can extract and represent the average intensity of multiple facial expressions in a crowd (Haberman & Whitney, 2007, 2009). This extraction occurs quickly and automatically even though participants are unable to recognize each face. A statistical summary is perceived from both sequentially presented single items and simultaneously presented multiple items (the latter is often called *ensemble perception*). In this paper, the term “ensemble” was used to describe the extraction based on multiple different intensities of facial expressions rather than the facial stimuli created by morphing software. In fact, as we often see multiple faces in our daily life, the recognition system of an ensemble of multiple faces is important for our daily communication. Although previous studies showed that individuals can extract an ensemble from multiple faces (Haberman & Whitney, 2007, 2009), the underlying mechanisms of this ability have not been fully revealed—for example, how multiple faces are represented in the minds of the participants. One possibility is that they extract and form visual information (or a visual representation) from multiple faces. However, individuals can possibly extract conceptual information (without a visual representation) from multiple faces, and this information affects their behavior. In this study, we examined the formation of a visual representation for an ensemble using a procedure of aftereffect (or adaptation) of facial expression.

The phenomenon of facial expression adaptation or aftereffect has been recognized as a useful tool for the evaluation of the recognition of faces (Webster, 2011). Previous studies have explored how adaptation to visual stimuli and its aftereffect can affect the perception of relatively low-level visual features, such as orientation and color (Gibson & Radner, 1937); however, these phenomena can also be used to assess their effect on the recognition of facial properties such as face identity (Leopold et al., 2001), gender (Webster et al., 2004), and facial expressions (Hsu & Young, 2004; Webster et al., 2004). Interestingly, adaptation to facial expressions does not require the initial facial expression (adaptation stimulus) and subsequent facial expressions (test stimulus) to be expressed by the same person (Fox & Barton, 2007). It is thus suitable to adopt the adaptation method to the situation of multiple face presentation. More importantly, the adaptation effects of facial expressions are considered to be related to visual processing (Fox & Barton, 2007), as the presentation of faces was found to induce an aftereffect of facial expressions, but emotional verbal and auditory stimuli were not. Moreover, Fox and Barton (2007) also showed that emotional but nonhuman facial (dog) stimuli induced a weak aftereffect. These results suggest that if the ensemble average is formed only by conceptual information of facial expressions, a weak or no aftereffect would be observed.

Nagy et al. (2012) demonstrated that adaptation to the gender of individual faces presented simultaneously distorted gender discrimination performance of a face subsequently presented; participants perceived an ambiguous test face as more masculine after adaptation to a female facial set and vice versa. The results were consistent with those of previous studies that used a single female or male face as adaptation stimuli (Kovács et al., 2008; Webster et al., 2004). This supports the claim that an ensemble extracted from multiple faces is represented using visualized information. However, the gender discrimination task is not enough to conclude this, because the gender of a face can be perceived based on either the arrangement of facial parts or the approximate size and shape of the face. For example, facial parts are closer to each other in female than in male faces, and female faces are smaller and rounder than male faces (Johnston, 2006). Therefore, participants can extract the average gender of faces without recognizing any detailed information about the faces (i.e., adaptation by a simple visual stimulus, such as in Corbett et al., 2012). In comparison, recognizing facial expressions requires individuals to assess the shapes of multiple facial parts and combine the information they provide (Calder et al., 2000).

As for multiple facial expression adaptation, Ying and Xu (2017) presented participants with multiple faces sequentially and showed perceptual bias: adapting to a happy face sequence biased the subsequently presented faces towards less happy. Their results showed that the magnitudes of the aftereffects were comparable with those for static faces that expressed the same intensity as the average face, indicating that the representation of multiple facial expressions may not involve only conceptual information, at least when they are presented sequentially. However, it remains unknown whether the same would apply if faces were presented simultaneously in different spatial locations.

In sum, in this study, we explored whether the average of multiple facial expressions presented simultaneously distorted participants’ recognition of facial expressions; our goal was to provide empirical evidence that observers can extract and form visual information from multiple faces in our minds. One additional consideration that should be noted is whether the aftereffect of multiple facial expressions has the same properties as that of a single facial expression. The single facial expression aftereffect has been observed to be robust in situations where the prior and subsequent facial expressions were from the same category (Hsu & Young, 2004; Juricevic & Webster, 2012). For example, participants were unable to perceive or recognize weakened happy facial expressions correctly after exposure to fully happy facial expressions, but they could recognize them after exposure to angry facial expressions. These results suggest the process of norm-based coding of facial expressions (Burton et al., 2015). To investigate whether an ensemble face extracted from simultaneously presented multiple faces had the same processing properties as a single face, we used both happy (Experiments 1, 2, and 4) and angry expressions (Experiment 3) as prior exposure to subsequent happy expressions.

In Experiments 1 and 2, the adaptation stimuli were four happy facial expressions presented simultaneously, all with different intensities of expression and different models. We compared the perception of facial expressions in the adaptation situation to that in the nonadaptation situation and hypothesized that biased perception in the adaptation situation would be found, but not in the nonadaptation situation. We also measured perceptual bias in two additional adaptation situations, one in which a single happy facial expression with the same intensity as the average of four faces was presented as an adaptation, and another in which there were four happy facial expressions of different individuals but of the same intensity as the average. Previous research indicated that the aftereffect of facial expressions occurs after a few seconds of exposure (Fox & Barton, 2007; Hsu & Young, 2004; Webster et al., 2004), but also after as little as 17 ms for an angry expression and 50 ms for a happy expression (Sou & Xu, 2019) after a facial expression was presented for 500 ms repeatedly (Moriya et al., 2013) and after 1 s (Burton et al., 2016). However, this aftereffect was not observed with a one-time presentation of 500 ms (Hsu & Young, 2004). These findings indicate that there is a minimum length of exposure required to elicit an aftereffect. Therefore, we presented multiple faces for 1,000 ms in the present study. In Experiment 3, angry facial expressions were presented as an adaptation stimulus instead of happy facial expressions. Moreover, in Experiment 4, 16 happy facial expressions were presented as adaptation stimuli to prevent participants from continuously focusing on individual faces.

## Experiment 1

In the first experiment, we examined whether the ensemble average of facial expressions would affect the recognition of subsequently presented facial expressions. We simultaneously presented four happy facial expressions of different models with varying intensities of happiness, followed by faces with subtle happy expressions. The perceptual bias to subsequent happy facial expressions was measured and compared across sessions.

### Method

#### Participants

Eleven Japanese undergraduate or graduate students (including five females; mean age = 20.8 years, *SD* = 1.3) with normal or corrected-to-normal vision participated. All participants were naïve to the purpose of the experiment. One of the participants was excluded from the analysis because her response of “happy” in the base session was noticeably low, with a point of subjective equality (PSE) higher than two standard deviations from the average of the other participants.

The number of participants required for the experiment was calculated in an a priori statistical power analysis using G*Power (Version 3.1.9.2; Faul et al., 2007) with *d* = .25 (medium-size effect), 1 − β = .95, and α = .05. The nearest even number of 10 was used for the sample size to make key assignments equal. This study was approved by the Ethics Committee in Unit for Advanced Studies of the Human Mind, Kyoto University (29-P-23). All participants provided informed consent before the experiment and received a monetary reward for their participation in the study

#### Apparatus

The experiment was conducted with a single participant in a dimly lit soundproof chamber. Stimuli were presented on a 19-inch DELL Trinitron P992 monitor (refresh rate 85 Hz, spatial resolution 1,024 × 768 pixels) using Windows 7 operating system and SuperLab 5.0.5 software (Cedrus) to design and control the experiment. The position of the participant’s head was fixed with a chin rest at a viewing distance of 45 cm.

#### Stimuli

**Fig. 1:**
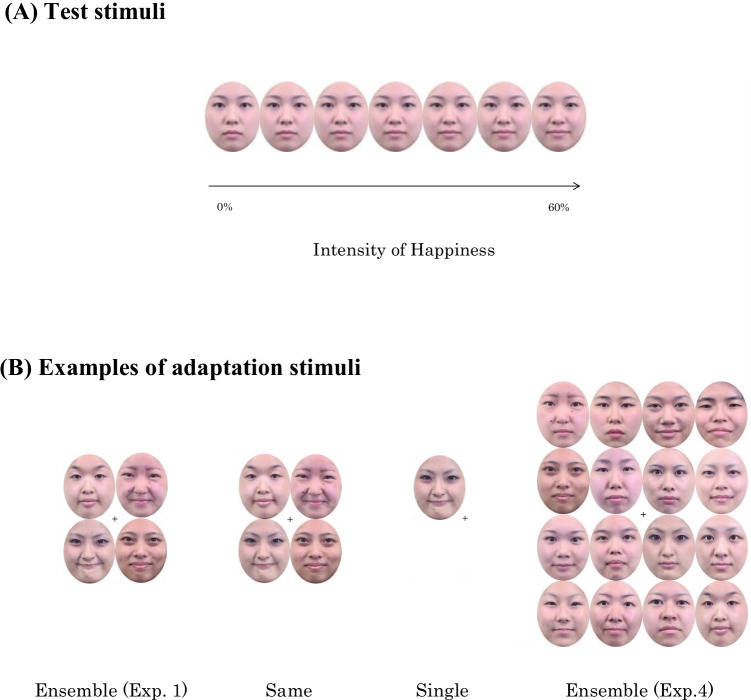
Test stimuli and adaptation stimuli used in Experiments 1–4. **(A)** Test stimuli; the 0% intensity of happiness (i.e., neutral face) was not presented in Experiments 2 and 4. **(B)** Examples of adaptation stimuli in the same, single, and ensemble conditions in Experiments 1 and 4. Eight photos in the ensemble condition in Experiment 4 were replaced from actual stimuli because of the portrait rights

The neutral and happy facial expressions of 35 Japanese female models were used. They were chosen from the Kokoro Research Center (KRC) facial expression database, which is a collection of photos of faces with expressions taken under controlled situations specifically for use in psychological experiments (Ueda et al., 2019), in which models were shown examples of facial expressions from Ekman and Friesen (1978) and instructed which action unit of the face should be moved to make an expression. Ueda and his colleagues reported that, for neutral and happy expressions, their stimuli were accurately perceived as expected facial expressions with evaluation and identification experiments. For adaptation stimuli, neutral and happy expressions of 10 out of 35 models were individually morphed to create happy expressions with intensities from 60% to 100% (in 10% increments). For test stimuli, neutral expressions of 35 models and happy expressions of 35 models were morphed, respectively, and these morphed neutral and happy expressions were further morphed. Thereby, happy expressions with intensities from 0% to 60% (in 10% increments) were created for the average of 35 faces (see Fig. 1A). A colored mosaic was created, based on the neutral face of the average of 35 individuals by shuffling pixels, and used as a mask. The sizes of all face stimuli and the mask were 2.4° × 3.2°. All face stimuli were cropped to an oval shape to exclude their hair and necks and presented in the center of the screen (test stimuli) or a square shape centered on the fixation point (adaptation stimuli). That is, the total size of the adaptation stimuli of four facial expressions was 4.8°× 6.4°, and the adaptation stimuli with one facial expression were presented in one of the spaces of the square shape.

#### Procedure

The experiment involved two sessions: a *base* (or *no-adaptation*) session and an *adaptation* session. These sessions were conducted separately, and the order was counterbalanced across participants. The participants’ task remained the same across sessions, which was to judge whether the test face had a happy expression or not by pressing the “F” or “J” key for “happy” or “not happy,” respectively. The key–response combination was counterbalanced across participants. Participants did not receive any feedback during the experiment. Each session started with 10 practice trials. The experiment stopped every 50 trials, and participants could take a break at that time.

**Fig. 2:**
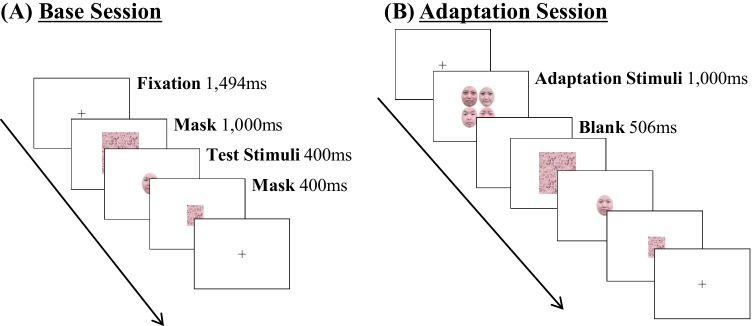
Trial sequences of Experiment 1. **(A)** Base session, in which no adaptation stimuli were presented. **(B)** Adaptation session, in which the adaptation stimuli were presented before the test stimuli. In this figure, we illustrated the ensemble condition in the adaptation session

***Base session*.** The sequence of trials is shown in Fig. 2A. Each trial began with the fixation cross for 1,494 ms, followed by the four masks presented simultaneously in a square shape centered on the fixation point for 1,000 ms. Subsequently, the test face was presented for 400 ms, followed by the mask for 400 ms. After the presentations, a blank screen with a fixation cross was presented until the participant responded. The next trial was initiated immediately after the response. Seven intensities of the test stimuli (0% to 60% in 10% increments) were presented 16 times each, for a total of 112 trials.

***Adaptation session.*** The sequence of trials for the adaptation session was the same as that for the base session, except that the adaptation stimuli were presented for 1,000 ms, and the blank screen was presented for 506 ms before the four masks were presented simultaneously in a square shape centered on the fixation point (see Fig. 2B). The adaptation time of 1,000 ms is long enough to elicit adaptation to facial expressions, according to Burton et al. (2016). There were three conditions for the adaptation stimuli (see Fig. 1B). In the ensemble condition, the happy facial expressions of 60%, 70%, 90%, and 100% intensity for different individuals were simultaneously presented at four locations around the fixation cross. In the “same” condition, four happy facial expressions of 80% intensity for different individuals were simultaneously presented at four locations around the fixation cross. In the single condition, one happy facial expression of 80% intensity (randomly selected for each trial from the adaptation faces) was presented at one of four locations around the fixation cross. Participants were explicitly instructed to maintain their focus on the fixation cross during the presentation of adaptation stimuli and to not focus on only one face.

In each of the three conditions, seven intensities of the test stimuli (0% to 60% in 10% increments) were presented, and each was repeated 16 times. Hence, the total number of trials was 336.

#### Analysis

For each condition, we conducted a probit analysis, in which the observed proportion of “happy” responses was fitted to a cumulative normal distribution function, and we calculated the average (mu) and standard deviation (sigma) for the function. The parameter “mu” indicated the PSE, at which the proportion of responses for “happy” and “not happy” were equal: this also means category boundaries between neutral and happy facial expressions. Additionally, we also calculated the just noticeable difference (JND), averaging the differences in stimulus intensity between 75% and 50% of “happy” responses and between 25% and 50%. A one-way repeated analysis of variance (ANOVA) was used to compare participants’ PSEs and JND among the four conditions (i.e., base, ensemble, same, and single). We also conducted Bayes factor ANOVA (JASP Team, 2019; Rouder et al., 2012) to estimate the likelihood of the null and alternative hypotheses. As there is no specific hypothesis, the prior settings of *r* scale fixed effects, random effects, and covariates were used in the default setting of JASP: 0.5, 1, 0.354, respectively.

### Results

**Fig. 3:**
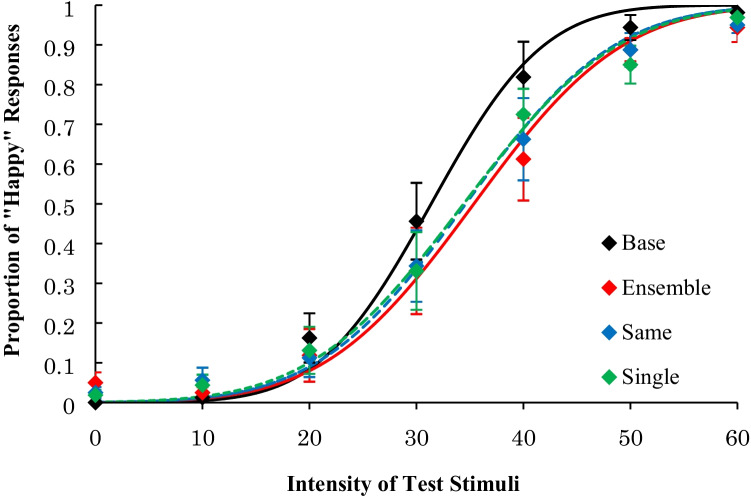
Results of Experiment 1: Psychometric functions of proportion of “happiness” response after adapting to happy faces

The average proportion of “happy” responses is shown in Fig. 3. The average PSE and the average JND across participants are shown in Table 1.

**Table 1:** Results of Experiment 1

	PSE (μ)		JND	
Base	31.38	(7.06)	5.57	(2.13)
Ensemble	35.37	(9.55)	7.37	(3.19)
Same	34.61	(9.05)	7.36	(2.37)
Single	34.42	(7.54)	7.61	(2.85)

*PSE =* point of subject equality, *JND =* just noticeable difference

For the PSE, there was a significant main effect, *F*(3, 27) = 4.45, *p* = .01, η_p_^2^ = .33. A multiple comparison procedure using Ryan’s method revealed that the PSEs for the three experimental conditions were higher than that of the base condition, *t*s(27) ≥ 2.58, *p* < .02, *r*s ≥ .44, and there were no significant differences among these three experimental conditions, *t*s(27) ≤ .81, *p* > .43, *r*s ≤ .15. Further, a Bayes factor ANOVA on the PSE with default prior scales revealed that the model including a main effect was preferred over the null model by a Bayes factor of 4.548. Post hoc comparisons indicated that the PSE in the base condition was lower than in other conditions (Bayes factors were 2.310, 2.789, and 3.425 for ensemble vs. base, same vs. base, and single vs. base, respectively), whereas no differences were observed between the three experimental conditions (Bayes factors were less than 0.417). These results indicate that the PSEs for the happy faces decreased after looking at the happy facial expression on one face, four faces with the same intensity, and four faces with different intensities compared with the PSE for the base condition.

For the JND, there was no significant main effect, *F*(3, 27) = 2.79, *p* = .06, η_p_^2^ = .24, suggesting that there were no differences in the dispersion of “happy” responses to each test stimulus among the four conditions. Further, a Bayes factor ANOVA on the JND with default prior scales revealed that the model including a main effect was preferred over the null model by a Bayes factor of 1.346. It is difficult to say that the data provide positive evidence for different effects of the adaptation presentations, given that the Bayes factor was relatively small.

### Discussion

We observed that the PSEs for a happy facial expression decreased after prolonged exposure to a happy facial expression on one face, four faces with the same intensity, and four faces with different intensities, compared with the PSE for the base condition. The decrease in the PSE was the same across the three adaptation conditions (i.e., ensemble, same, and single). These results suggest that the ensemble average of facial expressions, which was extracted from four different intensities and the facial expressions of four different persons, reduced the ability to recognize subsequently presented facial expressions to the same degree as when a single adaptation face was presented.

In Experiment 1, the intensities of the happy expressions in the adaptation stimuli were sufficiently strong to enable participants to identify their facial category, as they were over 60%. This finding suggests that one among four faces, rather than an extracted ensemble of four faces, may generate the adaptation effect in the ensemble condition. To test this possibility, we employed weaker facial expressions as adaptation stimuli in Experiment 2.

## Experiment 2

In the second experiment, we investigated whether extracting the average of all facial expressions had the same adaptation effect as a single facial expression. We presented two happy facial expressions of 20% intensity and two of 60% intensity (average 40%) as the adaptation stimuli in the ensemble condition and compared the degree of decreased PSEs for the happy facial expressions in this condition with those in a condition where a single face was presented with an intensity of 20% and 60% (the same as the intensities of the individual faces in the ensemble condition), and 40% (the same as the average intensities of the four faces in the ensemble condition). Previous aftereffect studies indicated that the degree of aftereffect depends on the intensity of the adaptation facial expressions (Hong & Yoon, 2018). Therefore, if participants extract average facial expressions from adaptation stimuli and adapt to them, their PSEs for happy facial expressions and dispersion of “happy” responses in the ensemble condition would be the same as those in the 40% condition. If not—that is, if they are adapted to only facial expressions with intensities of 20% or 60%—their PSEs for happy facial expressions would be close to those in either the 20% or 60% conditions. Conversely, if they randomly adapted to facial expressions with intensities of 20% or 60% in every trial, the dispersion of “happy” responses to each test stimulus in the ensemble condition would be higher than that in the other single presentation conditions. Although we utilized stepwise intensities in the ensemble condition in Experiment 1, we selected intensities of 20% and 60% in this experiment to clarify the difference in adaptation to weaker and stronger intensities and reduce the number of experimental conditions.

### Method

#### Participants

Eleven Japanese undergraduate or graduate students (including six females; mean age = 22.7 years, *SD* = 2.2) with normal or corrected-to-normal vision participated. All participants were naïve to the purpose of the experiment. One of the participants was excluded from the analysis because they were unable to complete the experiment due to technical difficulties.

#### Apparatus and analysis

The apparatus and analyses were identical to those in Experiment 1.

#### Stimuli

For the adaptation stimuli, we used the 10 models from Experiment 1 and created happy facial expressions of 20% and 40% intensity in the same way as in Experiment 1. In addition, the happy facial expressions of 60% intensity of each model from the stimulus set in Experiment 1 were also used. The test stimuli were the same as those in Experiment 1, as were the sizes of the stimuli.

#### Procedure

The procedure was the same as in Experiment 1, except for the conditions in the adaptation session and the number of repetitions. There were four conditions in the adaptation session: ensemble, single 20% intensity, single 40% intensity, and single 60% intensity. In the ensemble condition, two 20% and two 60% happy facial expressions of different models were simultaneously presented in the four locations around the fixation cross as the adaptation stimuli. In the single 20% intensity, single 40% intensity, and single 60% intensity conditions, one 20%, 40%, and 60% happy facial expression, respectively, was presented in one of the four locations around the fixation cross as the adaptation stimuli. In the base session, there were no adaptation stimuli. Each of the six intensity levels for the test stimuli (10% to 60% in 10% increments) was presented 14 times for a total of 84 trials in the base session and a total of 336 trials in the adaptation session.

### Results

**Fig. 4:**
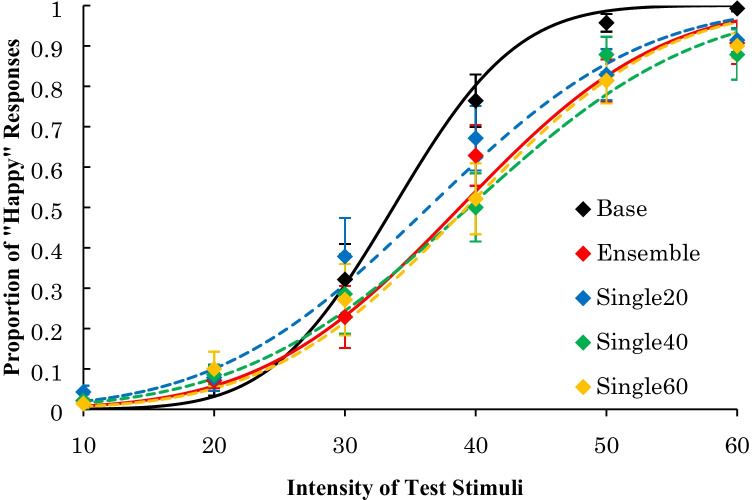
Results of Experiment 2: Psychometric functions of proportion of “happiness” response after adapting happy faces

The average proportion of “happy” responses is shown in Fig. 4. The average PSE and the average JND across participants are shown in Table 2.

**Table 2:** Results of Experiment 2

	PSE (μ)		JND	
Base	33.71	(5.48)	4.98	(3.17)
Ensemble	38.80	(6.86)	8.07	(4.45)
Single 20%	36.40	(6.88)	8.65	(5.73)
Single 40%	39.48	(7.64)	9.21	(7.93)
Single 60%	39.33	(7.90)	8.01	(4.63)

*PSE =* point of subject equality, *JND =* just noticeable difference

For the PSE, there was a significant main effect, *F*(4, 36) = 8.27, *p* < .001, η_p_^2^ = .48. A multiple comparison procedure using Ryan’s method revealed that the PSEs of the ensemble, single 40% intensity, and single 60% intensity conditions were higher than that of the base condition, *t*s(36) ≥ 4.19, *p*s < .01, *r*s ≥ .57. In addition, there were no significant differences among any of the other conditions, *t*s(36) ≤ 2.53, *p*s > .02, *r*s ≤ .39. Further, a Bayes factor ANOVA on the PSE with default prior scales also revealed that the model including a main effect was preferred over the null model by a Bayes factor of 269.739. This result provides extremely positive evidence of different effects of the adaptation presentations. Post-hoc comparisons revealed that the PSE in the base condition was lower than in other conditions (Bayes factors were 15.363, 4.431, 10.702, and 22.322 for ensemble vs. base, single 20% vs. base, single 40% vs. base, and single 60% vs. base, respectively). The PSE in the single 20% condition was also lower than in the ensemble, single 40%, and single 60% conditions (Bayes factors were 1.889, 2.717, and 15.280 for ensemble vs. single 20%, single 40% vs. single 20%, and single 60% vs. single 20%, respectively). The data did not provide evidence of differences between the ensemble, single 40%, and single 60% conditions (Bayes factors were less than 0.332). These results suggest that the PSEs for happy facial expressions decreased after looking at four happy facial expressions with an average intensity of 40% and a single happy facial expression with 40% and 60% intensity, but not after looking at a single happy facial expression with 20% intensity.

For the JND, there was no significant main effect, *F*(4, 36) = 2.38, *p* = .07, η_p_^2^ = .21, suggesting that there were no differences in the dispersion of “happy” responses to each test stimulus among the five conditions. Further, a Bayes factor ANOVA on the JND with default prior scales revealed that the model including a main effect was preferred over the null model by a Bayes factor of 1.092, suggesting that the positive evidence for different effects of the adaptation presentations was not strong.

### Discussion

The results from Experiment 2 indicated that the PSEs for the happy facial expressions in the ensemble condition were the same as those in the single 40% and 60% intensity conditions and higher than in the base condition. Moreover, the JND in the ensemble condition was not different from that in the other single presentation conditions.

Since the PSE in the base condition was comparable to that in the 20% intensity condition, if participants viewed facial expressions with intensities of 20% and 60% randomly in the adaptation, the dispersion of “happy” responses in the ensemble condition would be higher than that in the single presentation conditions. However, the results of the Bayes analysis suggest that the participants did not form representations from one of four adaptation faces presented randomly.

There were no significant differences between the PSEs of the ensemble condition and the single 60% condition, although participants did not observe one of the adapting facial expressions randomly; one possible explanation is that participants always searched for the face with the strongest intensity (60%). To prevent participants from using this strategy, the experimenter carefully instructed them to avoid focusing on only one facial expression before the experiment and, after the experiment, asked them to report how often their attention was captured by one of the facial expressions. Although some participants answered that their attention was captured when one face was presented, no participants answered that one face captured their attention when multiple facial expressions were presented, indicating that participants were not explicitly directing their attention to any single face. If participants viewed (or were attracted by) one face expressing the strongest expression after searching for it, the time viewing it would decrease, and the aftereffect would be weakened compared with the single 60% intensity condition.

## Experiment 3

The aftereffect of facial expressions is mainly observed when the adaptation and test facial expressions are from the same category of expression (Hsu & Young, 2004; Juricevic & Webster, 2012). To demonstrate the decrease in PSEs due to the facial expression aftereffect, we examined whether the adapted presentation of facial expressions different from happiness affected the recognition of test faces with happy expressions. Therefore, in Experiment 3, angry facial expressions were used as adaptation stimuli, and happy facial expressions were used as test stimuli.

### Method

#### Participants

Twelve Japanese undergraduate or graduate students (including five females; mean age = 23.5 years, *SD* = 5.1) with normal or corrected-to-normal vision participated. All participants were naïve to the purpose of the experiment. Two of the participants were excluded from analysis because their response of “happy” in the base session deviated significantly from that of the others, with PSEs that exceeded the average by a magnitude of more than two standard deviations.

#### Apparatus and analysis

Apparatus and analyses were identical to those in Experiment 1.

#### Stimuli

Using neutral and angry facial expressions of the same faces used in Experiment 1, we generated angry faces with weakened intensities for 10 models. As the adaptation stimuli, angry expressions with intensities from 60% to 100% (in 10% increments) were used. As the test stimuli, the same happy expressions with intensities from 0% to 60% (in 10% increments) from Experiment 1 were used. The sizes of the stimuli were also the same as in Experiments 1 and 2.

#### Procedure

The procedure was the same as in Experiment 1, except that angry rather than happy expressions were used as the adaptation stimuli.

### Results

**Fig. 5:**
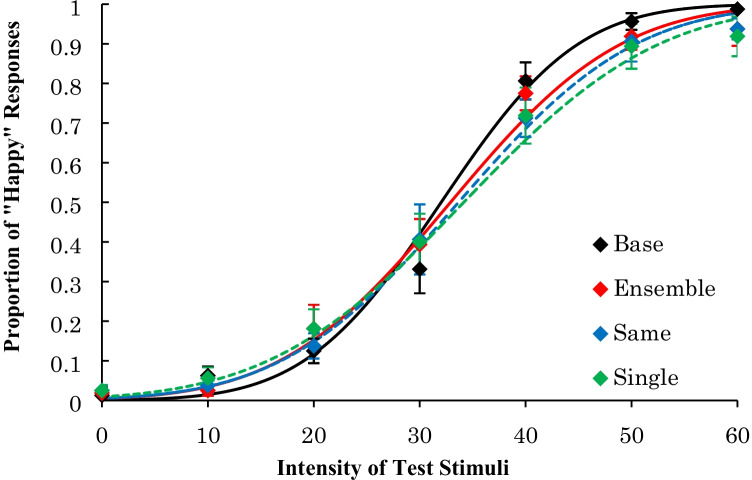
Results of Experiment 3: Psychometric functions of proportion of “happiness” response after adapting to angry faces

The average proportion of “happy” responses is shown in Fig. 5. The average PSE and the average JND across participants are shown in Table 3.

**Table 3:** Results of Experiment 3

	PSE (μ)		JND	
Base	32.03	(4.86)	6.83	(2.36)
Ensemble	32.91	(4.89)	8.50	(5.38)
Same	33.67	(5.51)	8.83	(4.16)
Single	34.16	(8.76)	9.74	(6.94)

*PSE =* point of subject equality, *JND =* just noticeable difference

For the PSE, there was no significant main effect, *F*(3, 27) = 0.65, *p* = .60, η_p_^2^ = .07, indicating that the PSEs for the happy faces did not decrease after looking at angry facial expressions of one face, four faces with the same intensity, or four faces with different intensities compared with the PSE of the base condition. Further, a Bayes factor ANOVA on the PSE with default prior scales revealed that the null model was preferred over the model, including a main effect by a Bayes factor of 0.241. This result provides substantial evidence for the null hypothesis.

Similarly, there was no main effect for the JND, *F*(3, 27) = 1.93, *p* = .15, η_p_^2^ = .18, suggesting that the presentation of angry facial expressions as the prime stimuli did not affect the dispersion of “happy” responses to each test stimulus. Further, a Bayes factor ANOVA on the JND with default prior scales revealed that the null model was preferred over the model, including a main effect by a Bayes factor of 0.661, providing substantial evidence for the null hypothesis.

### Discussion

The results of Experiment 3 indicated that the PSEs for happy facial expressions were not affected after the adaptation presentation of angry faces. These results suggest that the categories of facial expressions used in the adaptation and test stimuli are important for the decrease in PSEs, and the results observed in Experiments 1 and 2 were due to the aftereffect of the adapted presentation of the same facial expressions.

## Experiment 4

Experiments 1–3 showed that adaptation to multiple facial expressions of happiness biased the perception of subsequent happy facial expressions. However, in Experiments 1 and 2, four faces were presented for 1 s as an adaptation stimulus, and it is possible that participants easily recognized each individual or focused on the most salient face (i.e., with the strongest intensity). In Experiment 4, therefore, we used a larger set of faces as adaptation stimuli to confirm that participants adapted to the summary of multiple facial expressions (ensemble average), but not to the distinctive face (i.e., the face with the strongest intensity of facial expressions). Sixteen happy facial expressions that consisted of a mixture of the eight faces of 20% and 60% intensity, respectively (i.e., 40% on average), were presented as adaptation stimuli in the ensemble condition, and we investigated the adaptation effect on the recognition of subsequent happy facial expressions.

To make the difference in residual effects between conditions clearer and more stable, the following changes were applied. First, the base condition was always implemented first to provide participants with a reference point (Hong & Yoon, 2018). Second, there were three levels for the adaptation condition: single 20% intensity, ensemble (40% on average), and single 60% intensity. They were presented in separate blocks, and their order was counterbalanced across participants. Third, the number of repetitions was 20 for each test stimuli; therefore, the total trials were 480. Fourth, four practice trials were conducted before each block, and the experiment stopped every 60 trials for a break.

### Method

#### Participants

Eighteen Japanese undergraduate or graduate students (including seven females; mean age = 21.6 years, *SD* = 2.8) with normal or corrected-to-normal vision participated. All participants were naïve to the purpose of the experiment. The number of participants was calculated based on the difference between the results of the single 20% and ensemble conditions in Experiment 2 (*d* = .78, 1 − β = .80, and α = .05). This combination was chosen because it showed the largest difference relative to the ensemble condition. To counterbalance the allocation of keys and the order of the sessions, the number of participants was chosen to be 18, as it was the closest multiple of 6.

#### Apparatus

The apparatus was identical to those in Experiment 1 except that the experiment was operated via MATLAB (The MathWorks, Natick, MA, USA) and Psychtoolbox (Brainard, 1997; Pelli, 1997).

#### Stimuli

We used happy facial expressions with intensities of 20% and 60% of 16 individuals as adaptation stimuli. Ten individuals were the same as in Experiments 1–3, and six more individuals were selected from the models in the test stimuli. In the ensemble condition, they were presented with a 4 × 4 matrix with no gaps, and in the single 20% and 60% conditions, one face was presented in one of four locations closest to the fixation point. The test stimuli were the same as in Experiment 2. The sizes of the stimuli were also the same as in Experiments 1–3; thus, the sizes of adaptation stimuli in the ensemble condition and the mask after adaptation stimuli were 9.6° × 12.8°.

#### Procedure

There were some changes in the procedure relative to Experiment 2. We presented 16 faces, instead of four faces, in the ensemble condition. As mentioned at the beginning of the section on Experiment 4, four more points were changed to clear the difference between conditions.

#### Analysis

Analyses were identical to those in Experiment 2 except that only the three adaptation conditions were analyzed, as the base condition was always implemented first.

### Results

**Fig. 6:**
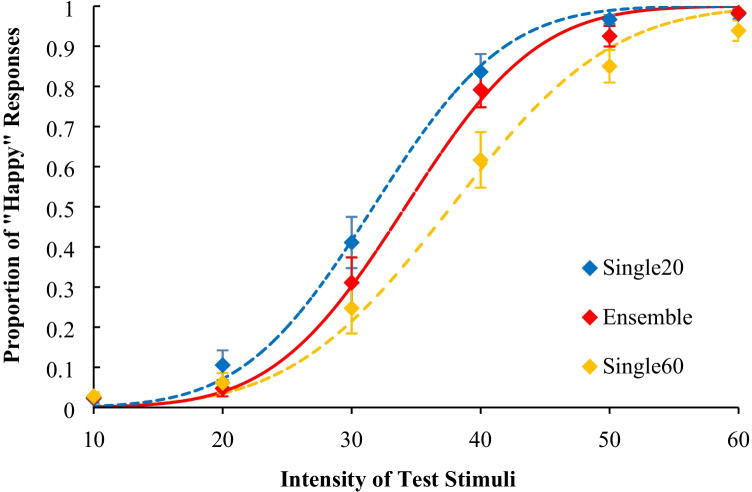
Results of Experiment 4: Psychometric functions of proportion of “happiness” response after adapting happy faces

The average proportion of “happy” responses is shown in Fig. 6. The average PSE and the average JND across participants are shown in Table 4.

**Table 4:** Results of Experiment 4

	PSE (μ)		JND	
Single 60%	37.72	(8.23)	6.59	(2.93)
Ensemble	34.17	(5.78)	5.44	(2.07)
Single 20%	31.69	(5.49)	5.37	(2.93)

*PSE =* point of subject equality, *JND =* just noticeable difference

For the PSE, there was a significant main effect, *F*(2, 34) = 9.62, *p* < .001, η_p_^2^ = .36. A multiple comparison procedure using Ryan’s method revealed that the PSEs of the single 60% intensity was higher than those of the ensemble and single 20% intensity condition, *t*s(34) ≥ 2.57, *p*s < .05, *r*s ≥ .40. There were no significant differences between the ensemble and single 20% intensity condition, *t*s(34) = 1.79, *p*s = .08, *r*s = .29. Further, a Bayes factor ANOVA on the PSE with default prior scales also revealed that the model including a main effect was preferred over the null model by a Bayes factor of 59.587. This result provides positive evidence for different effects between the adaptation conditions. Post hoc comparisons revealed that the PSEs in the ensemble and single 20% conditions were smaller than in the single 60% condition (Bayes factors were 1.641 and 29.694 for ensemble vs. 60% and 20% vs. 60%, respectively), and the PSE in the single 20% condition was also lower than in the ensemble condition (Bayes factor was 7.326). These data suggest that the PSEs for happy facial expressions decreased gradually according to the intensity of the adaptation stimulus in the ensemble and single conditions.

For the JND, there was a significant main effect, *F*(2, 34) = 4.86, *p* =.01, η_p_^2^ = .22. A multiple comparison procedure using Ryan’s method revealed that the JND of the single 60% intensity was higher than those of the ensemble and single 20% intensity condition, *t*s(34) ≥ 2.62, *p*s < .05, *r*s ≥ .41. There were no significant differences between the ensemble and single 20% intensity condition, *t*s(34) = 0.15, *p*s = .88, *r*s = .03. Further, a Bayes factor ANOVA on the JND with default prior scales revealed that the model, including a main effect, was preferred over the null model by a Bayes factor of 3.690. Post hoc comparisons revealed that the JNDs in the ensemble and single 20% conditions were smaller than in the single 60% condition (Bayes factors were 1.784 and 10.041 for ensemble vs. 60% and 20% vs. 60%, respectively). The data did not provide evidence of differences between the ensemble and single 20% conditions (Bayes factor was 0.246). These data suggest that the dispersion of the “happy” responses in the single 60% condition was higher than that in the ensemble and 20% conditions.

### Discussion

The results of Experiment 4 indicated that the PSE for happy facial expressions in the single 60% intensity condition was higher than in the ensemble condition, and further, the PSE in the ensemble condition was higher than in the single 20% intensity condition, suggesting that participants did represent ensembles with averaged intensity.

The JND in the single 60% intensity condition was larger than in the single 20% intensity condition, suggesting that the perceived impression of happiness might vary depending on the presented models in the single 60% condition. However, the JND in the ensemble condition was not different from that in the single 20% condition, suggesting that it is unlikely that participants randomly picked one face among the crowd and adapted to it. This is because if participants had adapted to one face with either 20% or 60% intensity randomly, the results in the ensemble condition would have been a mixture of the adaptation to faces with 20% and 60% intensity and the dispersion of these results (i.e., JND) would have been larger than those of single conditions. However, the results were not a mixture of adaptation to faces with 20% intensity and 60% intensity conditions. Furthermore, it is possible that this result is the average of the participants who adapted to a face with 20% intensity only and those who adapted to a face with 60% intensity only in the ensemble condition. If it were true, the results in the ensemble condition would have been the combination of individuals adapted to either 20% or 60% intensity, and the standard deviation of PSE would have been larger than those of single conditions, but the results showed that the standard deviation of PSE was not higher than 60% intensity, suggesting that this possibility also seems unlikely.

It is noted that we could not compare the adaptation conditions and base because the base phase was always conducted as the first session. This order was used to establish the standard of happiness for the participants before biasing by adaptation and to observe the difference between conditions clearly.

## General discussion

Four experiments were conducted to examine whether multiple facial expressions presented simultaneously as adaptation stimuli affected the recognition of facial expressions presented subsequently. We observed happy facial expressions as adaptation stimuli leading to the perceptional bias that makes it difficult to perceive happiness in test stimuli compared to the nonadaptation condition in Experiments 1 and 2. The effect was similar whether the adaptation stimuli were one face, four faces with the same intensity, or four faces with different intensities. In Experiments 2 and 4, we compared the PSEs and JNDs when a single face with a happy facial expression of either the strongest or weakest intensity was presented as the adaptation stimulus prior to the presentation of multiple faces that included both intensities. In Experiment 2, we observed that the PSE in the ensemble condition and the single 60% intensity condition (the strongest intensity of ensemble individual) was higher than that in the base condition. Alternatively, the PSE in the single 20% intensity condition (the weakest intensity of ensemble individual) was comparable to that in the base condition. For JNDs, there were no significant differences between the adaptation conditions. Furthermore, Experiment 4, with a larger face set (16 faces), showed that the PSEs gradually increased according to the intensities of the adaptation stimuli (i.e., single 20%, average 40%, and single 60%), and the JND in the ensemble condition was not different from that in other conditions; it was lower than in the single 60% condition. These results suggest that participants did not adapt to one of the facial expressions in the adaptation stimuli, but to the extracted ensemble average from the multiple facial expressions. In Experiment 3, we confirmed that the current results were observed only when the adaptation and test stimuli expressed similar facial expressions, indicating that the category of facial expressions of the adaptation and test stimuli is important in the ensemble facial expression aftereffect and was the same as the aftereffect of a single face.

To recognize ensemble facial expressions, unlike gender, it is necessary to assess the shapes of multiple facial parts and combine the information. Our results indicate that an ensemble average of facial expressions could be extracted from facial expressions presented simultaneously; thus, our findings are consistent with those of a previous study investigating gender (Nagy et al., 2012). In the ensemble conditions, the intensities of the individual facial expressions differed from their average intensity. For example, the average intensity was 80% in Experiment 1, but individuals showed facial expressions with 60%, 70%, 90%, and 100% intensities; the average intensity was 40% in Experiments 2 and 4, but individuals showed facial expressions with intensities of 20% and 60%. These results suggest that the participants represented the ensemble average of multiple facial expressions as real facial expressions. Although participants were not asked to extract the summary of the multiple faces and could not look at the actual averaged face, they adapted to the ensemble average. The results support the hypothesis that the ensemble is achieved instantaneously and automatically without much effort.

The results of this study provide empirical evidence that observers can extract and form visual information from multiple faces. Previous research indicates that adaptation effects are based on visual rather than conceptual representation (Fox & Barton, 2007). According to this, if the ensemble average used only conceptual information of happiness, the results in this study might have indicated a weak or no aftereffect. However, our results demonstrated that the aftereffect in the ensemble condition was the same as in other single face presentation conditions. Therefore, we can consider this the empirical evidence that ensemble faces would be represented in a visualized form. Since the adaptation procedure investigates only the visualized representation, our results do not deny that conceptual representations can be also formed via ensemble perception. The possibility remains that we simultaneously form visualized and conceptual representations of an ensemble average.

Our results revealed that participants’ perception of happy facial expressions was biased after viewing the same category of facial expressions. These results are consistent with those of previous research on adaptation (Hsu & Young, 2004; Juricevic & Webster, 2012; Webster et al., 2004) and suggest that the ensemble average of multiple facial expressions presented simultaneously also affects the recognition of facial expressions. Additionally, this category selectivity supports the norm-based coding of facial expressions (Burton et al., 2015). Our findings also indicate that the aftereffect of facial expressions occurs across identities: although faces selected from either 10 or 16 identities were used as adaptation stimuli, the average face of 35 identities was used as test stimuli. This indicates that we can process others’ facial expressions regardless of their identity, and this implication is consistent with previous research on aftereffects (Fox & Barton, 2007) and face models (Bruce & Young, 1986; Haxby et al., 2000).

We observed that the aftereffect of facial expressions occurred in more realistic situations compared with previous research. First, our results indicated that a duration of 1 s is sufficient to induce the aftereffect. The duration time of adaptation stimuli impacts the magnitude of the aftereffect: longer adaptation stimuli induce a stronger aftereffect logarithmically (Burton et al., 2016; Leopold et al., 2005; Mei et al., 2017; Rhodes et al., 2007). In a previous study, the duration time was 5 s or more, and it was often pointed out that this duration time was too long to elicit an aftereffect in daily life. Second, we used multiple and different intensities of facial expressions as adaptation stimuli, and the locations of adaptation and test stimuli varied. These situations are common in daily life.

Our results indicate that the magnitude of the aftereffect was comparable between the single face adaptation (single condition) and the four identical face adaptations (same condition). No previous study has investigated the effect of the number of facial expressions, but it is difficult to conclude that the aftereffects of facial expressions are not affected by the number of adaptation stimuli. One possibility is that our results were caused by a floor effect. For example, Moriya et al. (2013) demonstrated that using an adaptation paradigm, the perception of happy facial expressions changed more rapidly than that of angry facial expressions. In light of this finding, it may be that participants’ perceptual bias in this study was sufficiently decreased by the single face adaptation, and multiple presentations would not have reduced it further. The effect of the number of facial expressions on the aftereffect should be investigated using either another expression category or more subtle expressions in future research.

In this study, the results were congruent with the results of a previous study using sequential presentation (Ying & Xu, 2017), but it is unclear whether the representation of average is also the same. A theoretical model exploring this issue with facial attractiveness proposed that the temporal and spatial ensemble statistics are calculated differently (Ying et al., 2020). They used facial attractiveness because it is well known that the computer-based average face is more attractive than the individual faces composing the average due to averaging face textures and improving symmetry. It is beneficial to examine whether the process and representation of average is computer-like morph average or general gist average (simple average of each facial attractiveness). Their results showed that the spatial ensemble is extracted based on general gist average though the temporal ensemble is on morph average. Whether this theoretical model can be generalized to facial expression remains unclear because the averages calculated in two different ways (morph or gist) might be the same in facial expression. Considering the PSE results of Experiment 4, we can discuss the nature of the average representations. Though the morph steps between the 20% and 40% intensity faces and the 40% and 60% intensity faces were the same, the difference in proportions of happy responses between the 20% and 40% test faces was greater than between the 40% and 60% test faces. This result indicates the possibility that the average representation of 20% and 60% intensities might not be 40% rather than less if our average representations are not based on physical intensity, but on perceived intensity. This can also explain the results that the PSE difference between 20% intensity and the ensemble conditions was not significant though that between 60% intensity and the ensemble conditions was significant. Although we assumed that the average of stimuli was calculated based on physical intensity (that is, the average of the 20% and 60% intensity images was 40%), studies of color, for example, have reported a discrepancy between physical and perceptual intensity (e.g., Davidoff et al., 1999). This indicates a possibility that exact average of the 20% and 60% may be distorted from 40%. The precise intensity of representation formed by adaptation to multiple facial expressions can be examined in future research.

There are some other limitations to this study. First, we used only female models due to the number of faces in the database. In a study using the same stimulus set, there were no gender differences in dominance rating from expressions (Ueda & Yoshikawa, 2018), indicating that the difference in effects between male and female models might be small, but no studies have examined this in ensemble adaptation. This raises an open question regarding the generalizability of the results of this study (i.e., pertaining male faces or mixture of male and female faces). Second, we used no face presentation instead of neutral face presentation in the base condition. It is possible that perceiving faces (regardless of facial expression) biases the recognition of following facial expressions, but it is noted that we created test stimuli with neutral faces and happy facial expressions. If participants adapted to a neutral face, their perception of a neutral face would be suppressed, and the difference with happy adaptation condition would increase. To avoid this issue, we did not present any face stimuli in the base condition. However, the effect of neutral facial expression should be considered in future research.

In conclusion, the current study demonstrates that the ensemble average of multiple facial expressions presented simultaneously affects the recognition of facial expressions that are subsequently presented. We employed various models and intensities of expression as adaptation stimuli and showed that the magnitude of the aftereffect with an ensemble average of facial expressions was comparable to that with a single facial expression. These results support the idea that the representation of an ensemble of multiple facial expressions includes visualized information, not only conceptual information. Such aftereffect influences our cognition and may play a useful role in communicating within a group of multiple members.

## Supplementary Information

ESM 1 (DOCX 19 kb)
