# Supplementary material for: The aftereffect of the ensemble average of facial expressions on subsequent facial expression recognition
Source: Atten Percept Psychophys. 2022 Feb 15;84(3):815–28. doi: 10.3758/s13414-021-02407-w (PMC9001283; doi:10.3758/s13414-021-02407-w)
Supplement: Supplementary file 1 — (DOCX 19 kb) [file 13414_2021_2407_MOESM1_ESM.docx]

# Supplementary Information

Table S1. The point of subjective equality (PSE) for each participant in Experiment 4.

| Participant ID | 20% | | Ensemble | | 60% | |
| --- | --- | --- | --- | --- | --- | --- |
| 1 | 24.84 | (1.15) | 29.70 | (1.06) | 27.20 | (0.87) |
| 2 | 23.61 | (1.52) | 27.04 | (1.05) | 31.28 | (1.68) |
| 3 | 40.45 | (1.47) | 40.20 | (1.21) | 49.14 | (1.60) |
| 4 | 32.12 | (0.51) | 35.36 | (0.73) | 36.96 | (0.47) |
| 5 | 35.52 | (0.39) | 33.04 | (0.47) | 40.13 | (0.62) |
| 6 | 36.67 | (0.62) | 44.65 | (0.93) | 46.64 | (0.91) |
| 7 | 32.93 | (0.39) | 36.76 | (0.56) | 41.57 | (0.44) |
| 8 | 24.23 | (1.14) | 25.13 | (1.26) | 52.24 | (1.22) |
| 9 | 27.88 | (0.51) | 34.51 | (0.47) | 33.33 | (0.62) |
| 10 | 28.67 | (0.54) | 31.45 | (0.73) | 29.25 | (0.67) |
| 11 | 31.25 | (0.14) | 33.51 | (0.45) | 36.17 | (0.79) |
| 12 | 32.58 | (0.49) | 30.41 | (0.15) | 29.56 | (0.55) |
| 13 | 33.04 | (0.47) | 39.12 | (0.55) | 42.49 | (0.70) |
| 14 | 31.57 | (0.44) | 39.69 | (0.62) | 37.66 | (0.59) |
| 15 | 27.57 | (1.11) | 29.55 | (0.82) | 27.30 | (1.71) |
| 16 | 40.00 | (0.55) | 37.57 | (0.64) | 42.25 | (0.69) |
| 17 | 25.48 | (0.54) | 24.13 | (0.71) | 25.41 | (0.85) |
| 18 | 41.96 | (1.11) | 43.21 | (0.85) | 50.39 | (1.09) |
